# Socioeconomic status and self-rated health in Iran: findings from a general population study

**DOI:** 10.1186/s12962-022-00364-1

**Published:** 2022-06-29

**Authors:** Soraya Nouraei Motlagh, Zahra Asadi Piri, Heshmatollah Asadi, Razyeh Bajoulvand, Satar Rezaei

**Affiliations:** 1 Social Determinants of Health Research Center, Lorestan University of Medical Sciences, Khorramabad, Iran; 2 Ph.D Student in Health Care Management, School of Public Health, Tehran University of Medical Sciences, Tehran, Iran; 3 Department of Health Economics, School of Medicine, Shahed University, Tehran, Iran; 4 Student Research Committee, Iran University of Medical Sciences, Tehran, Iran; 5 Research Center for Environmental Determinants of Health, Health Institute, Kermanshah University of Medical Sciences, Kermanshah, Iran

**Keywords:** Health equity, Socioeconomic factors, Health-related quality of life, Iran

## Abstract

**Background:**

There are large gaps in health and well-being among different groups of the society. Socioeconomic factors play a significant role in determining the health status of the society. The present study was conducted to examine socioeconomic inequality in health status among the adult population of Khorramabad city, the capital of Lorestan province, wester part of Iran.

**Methods:**

A cross-sectional study was conducted on 1348 participants selected through multistage sampling. A valid and reliable questionnaire was used for data collection. The wealth index as an indicator of the socioeconomic status (SES) was used to categorize the subjects in terms of the SES. The concentration index and concentration curve was used to measure socioeconomic inequity in poor self-rated health (SRH) of population. Finally, after determine the status of inequity in poor SRH, a decomposition analysis approach was used to identify the most important determinants of this inequity.

**Results:**

The prevalence of poor SRH was 18.91% in all subjects, 38.52% in the lowest SES group, and 11.15% in the highest SES group. The value of the concentration index for poor SRH was − 0.3243 (95% CI − 0.3996 to − 0.2490), indicating that poor SRH was more concentrated among the poor. The results of decomposition analysis showed that SES (41.2%), higher body mass index (28.6%) and lack of physical activity (26.9%) were the most important factors associated with the concentration of poor SRH in the poor groups.

**Conclusion:**

Identification of socioeconomic factors affecting on health status is the first step for proper policymaking. Policymakers and health system managers at the national and subnational levels can use the results of this study as well as other similar domestic studies to design and implement proper interventions to promote equity and improve the health status of population.

## Introduction

Quality of life is now a very important aspect of public health and is a challenge for researchers in the present century [1]. The WHO defines quality of life as an individual's perception of their position in life, goals, expectations, standards, and priorities in the context of the culture and value systems in which they live [2]. Health is the core of quality of life [3]. Since the quality of life cannot be comprehensively addressed in the health system, the concept of health in relation to the quality of life is studied, which is defined as “health related quality of life” (HRQoL) [4]. One of the way to measure HRQoL is Self-rated health (SRH). SRH is an important objective and indictor of providing healthcare services to different groups of the society [3, 5]. HRQoL is a subset of overall life quality, and includes domains of mental, emotional, social, and physical well-being and reflects the mental assessment of patients and their response to the disease [6]. HRQoL evaluates the relationship between health status and quality of life (QoL) systematically; moreover, it is considered an important indicator of the outcomes of treatment and care interventions in diseases [7–9].

HRQoL is an important indicator for comprehensive measurement of health status and is increasingly used to measure health inequity among different social groups [10]. Health inequity among different social groups is major public health concern [11]. Although life expectancy and healthy life expectancy have improved globally in recent decades, this improvement has been associated with inequity and there is a large gap in health and well-being between the poor and rich. In other words, there health inequity between and within countries, for example, there is a difference of 18 years in life expectancy between high- and low-income countries [12]. Moreover, these inequities exist in a wide spectrum of health interventions as well as many social status indicators like Wealth, income, and education [13]. However, social determinants of health have an important role in the equal distribution of health outcomes among people [14, 15]. Social inequalities in health may result from differences in healthy behaviors and/or inequality in access to healthcare services in different groups [11]. Several studies have shown a significant relationship between socioeconomic status) SES (and morbidity and mortality [16, 17]. Most of the studies have confirmed that SES is the most important determinant of health, because it affects health through different mechanisms [15, 18]. Evidence shows that socioeconomic factors determine 30–55% of health-related outcomes [19]. People in low SES groups are more likely to have unhealthy habits and face more socioeconomic pressure; moreover, SES has a close relationship with the quality and quantity of healthcare services [20]. Moreover, socioeconomic factors are an important determinant of quality of life [21]. In the past decade, socioeconomic factors and their relationship with quality of life were interesting topic for research in the field of health [18]. Therefore, measurement of inequities in health requires knowledge about health and SES at an individual level. Some studies used data at an individual level to evaluate inequities and investigate causality between SES and health inequity [22]. In two studies conducted in Iran, the results showed that socio-economic status (45.5) and (69.44) were the most important factors of inequality in quality of life related to health, respectively [23, 24]. Also in China, various studies have reported that socio-economic status is a major cause of health inequality resulting from avoidable factors is a type of health inequity, which needs to be eliminated or alleviated through policy optimization [18, 24, 25]. Moreover, ensuring equal distribution of SRH among different groups and regions is important for policymakers. Therefore, it is necessary to measure and identify the determinants of SRH to design and implement effective policies to decrease social inequalities in health and the related quality of health [26–28]. Population-based studies aiming at assessing the level and determinants of the quality of life can potentially provide valuable information about SRH in different socioeconomic groups [27]. Therefore, since no study has evaluated the SRH and its relationship with the distribution of socioeconomic factors in Lorestan province, thus to fill this gap in the literature, we used the concentration index approach to measure socioeconomic-related inequalities in poor-SRH in adults in Khorramabad city. We also did a decomposition analysis of socioeconomic inequality in poor SRH to determine main factors affecting on the observed inequality in health.

## Methods

A cross-sectional study was conducted in Khorramabad, Lorestan province in 2019. The data was collected using multistage sampling. In the first stage, all urban health centers and all health stations of Khorramabad were defined as strata, and the households covered by these centers were determined. Then, the sample size in each stratum was determined using the allocation method proportional to the share of the population. In the second stage, in each stratum, the households were selected with random sampling using the patient record number. The sample size was calculated according to the following formula:

$$\mathrm{n}= \frac{{\mathrm{Z}}^{2} (\mathrm{p}\left(1-\mathrm{p}\right))}{{\mathrm{d}}^{2}}$$

Considering a poor SRH to reach a maximum sample size (0.5) with a confidence interval of 95% and precision of 0.03, a sample size of 1070 was calculated. Subsequently, to improve generalizability, the calculated sample was increased by 25% and finally 1347 subjects were included in the study [23].

The inclusion criteria were living in Khorramabad city for at least one year and age above 18 years. The only exclusion criterion was hesitation to join the study. At the household level, the first informed person aged above 18 years who was willing and able to answer the questions completed the questionnaire.

A questionnaire with confirmed validity and reliability was used for data collection [23, 28]. This questionnaire had two sections. The first section contained questions on demographics (age, sex, marital status), SES (education level, wealth index, having medical insurance), and lifestyle related factors (smoking, physical activity, BMI, chronic diseases) [23, 28], which were considered as explanatory variables of the quality of life.

The self-rated health (SRH) approach was used to assess the HRQoL, which is a valid and common indicator for health status evaluation. In this approach, the participants were asked to evaluate their current health status on a 5-point Likert scale from very good (5) to very bad (1) [29, 30]. In the next step, the health status was categorized into poor and good. Good health status included subjects who rated their health as very good and good, and poor health status included subjects who rated their health as average, bad, and very bad.

In line with other studies [23, 31], the wealth index was used as an indicator of SES to group the participants according to the SES. To build the wealth index, the data of household assets that had a stronger relationship with the household’s wealth level (number of rooms per person in the household, area of the house, type of property ownership, car, desktop computer laptop computer, dishwasher, washing machine, fridge, etc.) were collected and analyzed. In the second step, principal component analysis (PCA) was used to identify the variables that had larger effects on the variance of all variables, and more important variables were determined [32–34]. As per wealth scores, the households were divided into five groups, including the poorest, poorer, middle, richer, and richest.

The concentration index was used as a measure of socioeconomic inequality in poor SRH. The concentration curve (CC) is used to measure the concentration index, which is defined as twice the area between the concentration curve and the line of equality (a 45-degree line). CC ranges from -1 to + 1. If there is no inequality in the quality of life between socioeconomic groups, the CC will be a 45-degree line and the value of the concentration index will be zero. If CC is above the line of equity, it indicates that the concentration index has a negative value and poor SRH is concentrated in the non-affluent group. On the contrary, if CC falls below the line of equity, it indicates that the concentration index has a positive value and poor SRH is concentrated in the affluent group [24, 35, 36]. Concentration index was calculated using the following formula (37, 38):

$$\mathrm{CI}\hspace{0.17em}=\hspace{0.17em}\frac{2*cov{{(y}_{i}r}_{i})}{\mu }$$

Where ${\mathrm{y}}_{i}$ is the outcome variable in the ${\mathrm{i}}_{th}$ person, ${r}_{i}$ shows the fractional rank in the SES distribution for the ${i}_{th}$ person in the sample and *µ* the mean of the outcome variable.

Since the outcome variable is a binomial variable, the value of the concentration index will range between + 1 and − 1; instead, the value of the concentration index will be normalized by dividing the calculated value by $\frac{1}{1-\mu }$ [39]. After determining the status of inequality in poor SRH, a decomposition analysis approach was used to identify the most important determinants of inequality. For this reason, logistic regression analysis was applied to determine partial effects of independent variables on poor SRH as a binomial dependent variable (poor SRH is 1 and others zero). Moreover, age, sex, marital status, health insurance coverage status, presence or absence of chronic diseases, SES, smoking, obesity, were physical activity were entered into the model as explanatory variables. The formula suggested by Wagstaff et al. was used to determine the contribution of independent variables to inequality in poor SRH [40].

$$\mathrm{CI}\hspace{0.17em}=\hspace{0.17em}\sum_{K}\left(\frac{{\beta }_{k}{\overline{X} }_{k}}{\mu }\right){CI}_{k}+\frac{{GC}_{\varepsilon }}{\mu }$$

First, the beta coefficient of each independent variable was multiplied by its mean value (${\overline{\mathrm{X}} }_{k}$) and the result was divided by the mean value of the outcome variable. The obtained value indicated elasticity ($\frac{{\upbeta }_{k}{\overline{\mathrm{X}} }_{k}}{\mu }$). In the next step, the concentration index (${\mathrm{CI}}_{k}$) was calculated for each independent variable and the obtained value was multiplied by elasticity to determine its contribution to the concentration index. If the contribution of an explanatory variable took a positive (negative) value, it indicated that that socioeconomic distribution of this variable and its relationship with poor SRH resulted in the concentration of poor SRH among the poor (rich). $\frac{{\mathrm{GC}}_{\upvarepsilon }}{\upmu }$ Shows the residual component and reflects socioeconomic inequalities related to poor SRH not otherwise explained by explanatory variables entered into the model. The Stata software version 14 was used for data analysis.

## Results

The total prevalence of poor SRH was 18.91% (95% CI 16.8 to 21.01%). The mean ± SD age of the participants was 38.8 ± 13.3 years. Of 1348 adults aged 18–65 years, 47.8% were male, 67.28% were married, about 12% had at least one chronic disease, 32.32% had a less than high school diploma education, 74.33% had insurance coverage, and 53.93% had a normal BMI. In terms of education level, the total prevalence of poor SRH was 48.98% in illiterate subjects, while its prevalence in the highest education group was 12.61% (p < 0.001). In terms of age, the total prevalence of poor SRH ranged between 7.87% in the age group 18–30 years to 60.22% in subjects over 60 years (p < 0.001). Moreover, 14.6% of men and 22.87% of women had poor SRH (p < 0.001). The prevalence of poor SRH was 19.96% and 15.9% in subjects with and without insurance coverage, respectively. The prevalence of poor SRH was 14.72%, 19.6%, and 41.32% in normal weight, overweight, and obese participants, respectively (p < 0.001). The prevalence of poor SRH was 6.2% in physically active and 47.62% in physically inactive subjects. Among different SES groups, the prevalence of poor SRH ranged between 38.52% in the lowest SES group and 11.15% in the highest SES group (p < 0.001). The prevalence of poor SRH was significantly higher in subjects with at least one chronic disease (67.5%) compared to others (12.37%) (Table 1).

**Table 1:** Summary descriptive of the study samples

Explanatory variables	good SRH		poor SRH		P-value
	N	%	N	%	
Age groups					< 0.001
18–30	398	92.13	34	7.87	
31–45 years	460	85.34	79	14.66	
46–60 years	198	69.72	86	30.28	
61 years and above	37	399.78	56	60.22	
Gender					< 0.001
Male	550	85.4	94	14.6	
Female	543	77.13	161	22.87	
Marital status					
Single	321	90.17	35	9.83	< 0.001
Married	726	80.04	181	19.96	
Other	46	54.12	39	45.88	
Education level					< 0.001
Illiterate	50	51.02	48	48.98	
Less than diploma	246	72.78	92	27.22	
Diploma and bachelor’s degree	700	87.39	101	12.61	
Master’s degree and higher	97	87.39	14	12.61	
Health insurance					< 0.001
Yes	802	80.04	200	19.96	
No	291	84.1	55	15.9	
Chronic condition					< 0.001
Yes	52	32.5	108	67.5	
No	1041	87.63	147	12.37	
Smoking status					< 0.001
Never	970	82.41	207	17.59	
Former	50	62.5	30	37.5	
Current	73	80.22	18	19.78	
BMI					< 0.001
Normal	620	85.28	107	14.72	
Overweight	402	80.4	98	19.6	
Obesity	71	58.68	50	41.32	
Physical activity					< 0.001
Good	635	93.8	42	6.20	
Moderate	315	79.15	83	20.85	
Weak	143	52.38	130	47.62	
Socioeconomic status					< 0.001
Poorest	166	61.48	104	38.52	
Poorer	219	81.11	51	18.89	
Middle	230	85.5	39	14.5	
Richer	239	88.52	31	11.48	
Richest	239	88.85	30	11.15	

**Fig. 1:**
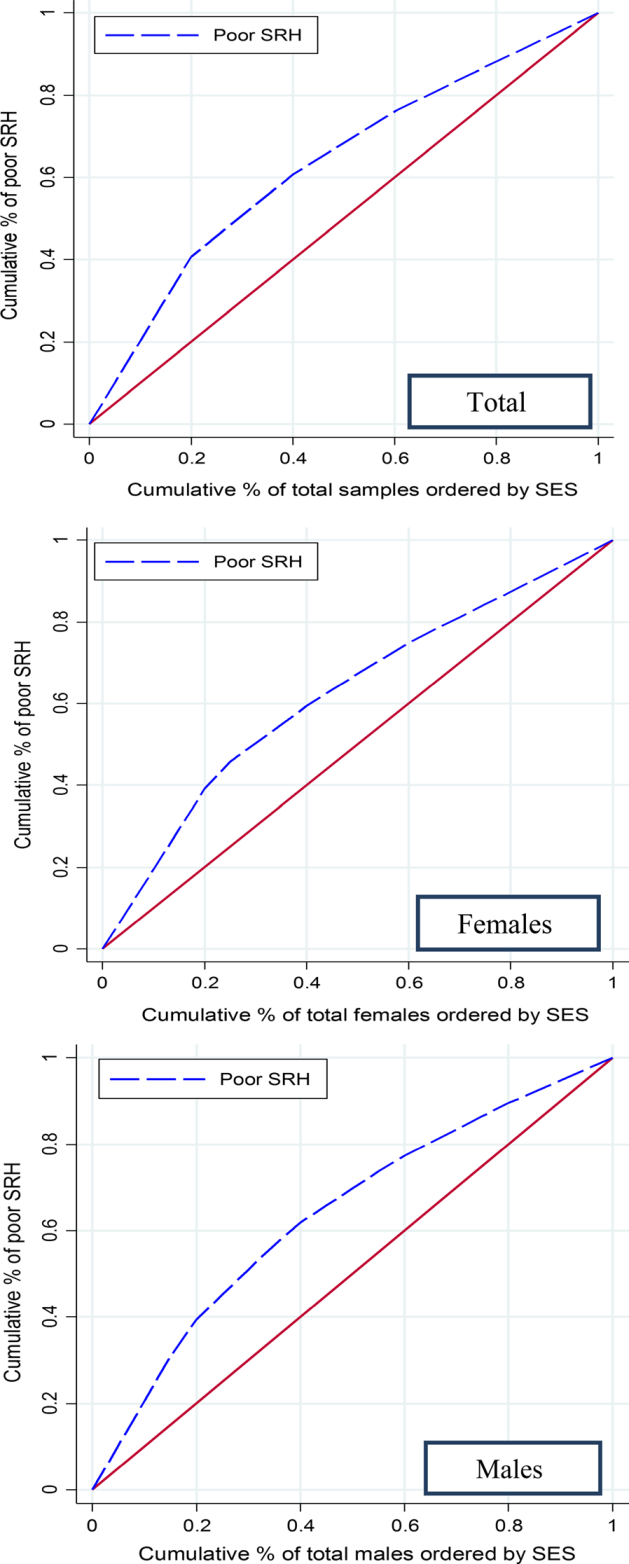
Concentration curve for poor self-rated health (SRH) in males, females and in total sample

The results of the concentration index showed poor SRH was more prevalent in people in people with lower SES (− 0.3243). The results of the concentration index in men and women were similar to the whole sample. The results of the concentration curve in men, women, and all samples are presented in Fig. 1. The concentration curve of poor SRH was above the 45-degree line for men, women, and all samples, indicating that poor SRH was concentrated in the poor (see Table 2).

**Table 2:** Normalized concentration index for poor self-rated health in Khorramabad, Lorestan Province, 2020

	Relative concentration index	Confidence interval 95%	p-value
Female	− 0.3207	− 0.4176 to − 0.2237	< 0.0001
Male	− 0.3259	− 0.4475 to − 0.2044	< 0.0001
Whole of sample	− 0.3243	− 0.3996 to − 0.2490	< 0.0001

Table 3 shows the contribution of explanatory variables to socioeconomic inequalities in poor SRH. The significant positive value of the final coefficients in older age groups shows a relationship between older age and increased probability of poor SRH. Compared to men, the probability of poor SRH was higher in women. A lower SES was associated with a higher probability of poor SRH in adults. Smoking, lack of physical activity, higher BMI, and having a chronic disease were associated with higher probability of poor SRH. The concentration index for each explanatory variable (${C}_{k}$) is presented in the third column of Table 3. A positive value of this index indicates that the explanatory variable of interest was concentrated in the rich and vice versa. The results (${C}_{k}$) showed that variables like older age, female gender, less than high school diploma education, lack of insurance coverage, having a chronic disease, being a former or current smoker, obesity, and moderate and weak physical activity were concentrated in the poor. Wealth index (41.2%) had the highest contribution to socioeconomic inequalities in poor SRH. In addition to wealth, BMI and physical activity were other important determinants of this inequality. The negative contribution of chronic disease, female gender, lack of physical activity, and smoking to socioeconomic inequalities in poor SRH indicated that the socioeconomic distribution of these variables in adults in the study population and their relationship with poor SRH resulted in the concentration of poor SRH among adults with a lower SES.

**Table 3:** Decomposition of socioeconomic inequalities in poor SRH among Iranian adults

	Marginal effect	Elasticity	*C*_*x*_	Contribution to the *C*_*n*_		
				Contribution	%	Summed%
Demographic variables						
Age Group (18–30 [ref.])						
31–45 years	0.039	0.082	− 0.035	− 0.004	1.1	
46–60 years	0.127	0.141	0.049	0.008	− 2.6	
61 years and above	0.186	0.068	− 0.039	− 0.026	8.0	6.4
Gender (Male [ref.])						
Female	0.064	0.178	− 0.009	− 0.002	0.6	0.6
Marital status (Single [ref.])						
Married	− 0.017	− 0.06	− 0.014	0.001	− 0.3	
Other (Divorce, separated and widows)	0.029	0.01	− 0.282	− 0.003	1	0.7
Socioeconomic variables						
Education status (illiterate [ref.])						
less than diploma	0.007	0.009	− 0.223	− 0.002	0.8	
High school diploma and bachelor’s degree	0.017	0.052	0.091	0.006	− 1.8	
Master’s degree and above	0.011	0.005	0.360	0.002	− 0.7	− 1.7
Wealth index of individuals (Poorest [ref.])						
Poorer	− 0.100	− 0.106	− 0.399	0.052	− 16.1	
Middle	− 0.123	− 0.130	0.001	0.000	0.0	
Richer	− 0.111	− 0.119	0.401	− 0.059	18.1	
Richest	− 0.121	− 0.128	0.801	− 0.127	39.1	41.2
Health insurance status (Yes [ref.])						
No	− 0.0033	− 0.045	− 0.189	0.010	− 3.2	− 3.2
Life style variables						
Chronic condition (No [ref.])						
Yes	− 0.216	− 0.136	− 0.118	− 0.020	6.1	6.1
Smoking status (Never [ref.])						
Former	0.087	0.027	− 0.128	− 0.004	1.3	
Current	0.055	0.020	− 0.205	− 0.005	1.5	2.8
BMI (Normal [ref.])						
Overweight	0.003	0.006	0.023	0.023	− 7.2	
Obesity	0.122	0.058	− 0.116	− 0.116	35.8	28.6
Physical activity (Good [ref.])						
Moderate	0.101	0.158	− 0.056	− 0.034	10.4	
Weak	0.234	0.251	− 0.173	− 0.054	16.5	26.9

## Discussion

Quality of life measurement in the general population is an important issue for health policymakers and is necessary to develop proper intervention aiming at quality of life improvement. The present study was conducted to identify socioeconomic-related inequalities in poor SRH in the adult population of Khorramabad in 2019. The use of SRH as a simple measure both in survey and clinical settings to identify vulnerable older adults and according to the evidences the validity of SRH is increasing. According to the results, the prevalence of poor SRH was 38.52% in the lowest and 11.15% in the highest SES group. A low SES had a significant relationship with poor SRH. According to the concentration curve and index, poor SRH was concentrated in the poor in males, females, and all samples. In a study in China, concentration index of SRH was 0.06, indicating a health disparity in favor of the rich [26].In 2012, a study in Turkey found that the poor SRH concentration index was − 0.15, suggesting inequality in the SRH, and that the poor SRH was more likely to be found in the poorer people [41]. According to another study in Tehran, poor SRH was more concentrated among the poor (concentration index = − 0.29) [31].

The results of the decomposition of socioeconomic-related inequalities in poor SRH showed that wealth was the most important contributor to inequality. There are several reasons for the negative relationship between SES (as indicated by wealth index) and poor SRH. SES determines the work and living environment of people and their access to different services and products [42]. Previous studies in Iran found higher utilization of health services in people from high SES groups compared to those from low SES groups [33, 34]. In addition, SES affects the mental state of people and their cognition of the surrounding world [43, 44]. Income is often considered an important predictor of health, and income inequalities are an important risk factor that can negatively affect health outcomes; therefore, re-distribution policies that reduce income inequalities, in addition to reducing inequalities in health outcomes, can also decrease inequalities in other social outcomes as well [45]. In line with the results of this study, a study conducted in the Chinese general population showed that a high SES had a positive relationship with quality of life) the concentration index of the EQ-5D and VAS indices were 0.022 and 0.026 respectively [18].

A study in Chile also found a negative relationship between SES and poor HRQoL in adults over 25 years of age [46]. The positive value of the concentration index in a study in China showed that rich people reported fewer health problems and had a better HRQoL compared to the poor [25].

The results of the decomposition analysis of socioeconomic-related inequalities in poor SRH showed that after wealth, other main determinants of socioeconomic-related inequalities in health were the presence of chronic disease, lack of physical activity, and BMI. In other words, the presence of chronic disease, lack of physical activity, and higher prevalence of obesity in the poor lead to the higher concentration of poor SRH among the poor. A study by Djärv et al. in Sweden also showed that a larger number of chronic diseases and lack of physical activity were the most important determinants of HRQoL [10]. Studies conducted in Iran also showed that chronic diseases were an important determinant of QoL in Iran, and prevention and management of chronic diseases was a priority to improve the HRQoL of Iranians [47, 48]. A study in England also found a relationship between low physical activity poor HRQoL [49]. It has been reported that high physical activity has a negative relationship with poor HRQoL [50]. Rezaei et al. found that physical activity explained more than 14% of inequality in poor HRQoL [23]. Rezaeipandari et al. reported that among different aspect of quality of life, physical activity had the strongest correlation with QoL [51]. According to Feizi et al., physical activity not only had a positive effect on the physical dimension of QoL, but also has positive impacts on other dimensions [52]. Ramezani et al. found that a marked percentage of Iranian people (65%) had no physical activity [45]; therefore, designating and implementing proper interventions to improve the level of physical activity in adults may lead to improved health status and health outcomes in this population.

As mentioned earlier, the higher prevalence of obesity in the poor resulted in the higher concentration of poor SRH in this group. Other studies have reported lower utility scores for subjects with a high or low BMI [53, 54]. Hajian-Tilaki et al. found a negative relationship between HRQoL and BMI [55]. A higher prevalence of obesity in the rich led to a higher concentration of poor SRH in this SES group [50].

Similar to other studies [49, 56], poor HRQoL was more prevalent in women. An explanation for the lower QoL in women could be a higher prevalence of anxiety and depression symptoms in Iranian women [57, 58]. In addition, this finding may be due differences in the economic status and social position between men and women [59]. Women are more likely to experience multiple roles, and other people’s expectations of women in each role may be different from their expectations or be contrary to their goals [60]. In line with other studies [25], the results of the present study showed that education level was an important factor that can explain inequality in poor HRQoL in favor of the poor (rich people have higher education levels compared to the poor). A cross-sectional study in Switzerland found a prevalence of 43% for poor health-related quality of life in people with lower education levels (less than 9 years) while the prevalence of 26% in people with higher education levels (more than 12 years) [48]. A lower education level is associated with weaker social activities, less cheerfulness, and lower self-esteem, which reduced the quality of life. Adult people with higher education levels are more aware of preventive measures regarding conditions and chronic diseases like diabetes, heart disease, cancer, and MI [61]. Evidence suggests that chronic diseases are more prevalent in people with lower education levels [62].

In the present study, having insurance coverage resulted in the concentration of poor SRH among the rich. A study in the United States found that people lacking insurance coverage had higher scores of the HRQoL-PCS compared to those covered by Medicaid and Medicare. This difference may be due to the health status of people, not the effect of access to healthcare services facilitated by medical insurance. Therefore, adjusting for the effect of health status can further clear the relationship between medical insurance and HRQoL [63]. Previous studies conducted in Iran found a positive relationship between having medical insurance and HRQoL [28, 50]. However, another study in Iran showed that having medical insurance had no significant effect on the poor HRQoL [38]; therefore, more studies are required to determine the relationship between HRQoL and having health insurance.

According to the results of the present study, the prevalence of poor HRQoL increased with age, which is consistent with previous studies [64]. The ageing process worsens mental health and cognitive disorders [65, 66]. The quality of life does not reduce merely as a result of ageing, and isolation and reduced social activities are also involved [67]. The Iranian population is aging [68], which causes challenges in improving the quality of life of the elderly population.

In line with a study conducted by Kazemi Karyani et al. [38], the prevalence of poor SRH was higher in the “other group (divorced, separated and widowed/widower)” compared to married and single subjects. Several studies have evaluated the relationship between marital status and SRH. However, the results are inconsistent and contradictory. A study conducted in Ethiopia showed that married people who lived separately had lower QoL scores compared to couples that lived together [69]. In another study, patients who lived with their partners had higher QoL scores compared to patients that lived alone, the difference in the QoL score was explained by other factors like SES, sex, and age, not by marital status, in the multiple model [70].

In the present study, smokers reported a worse health status compared to non-smokers. A study conducted in Kermanshah, Iran found a negative relationship between smoking and HRQoL [71]. In another study conducted in Iran, smokers had a lower QoL in physical, environmental, social, and psychological domains of health [72]. Moreover, a study found that smoking had a negative relationship with HRQoL in the general population of England [73]. These findings underline the importance of public education with emphasis on the harms of smoking and benefits of smoking cessation [74]. In fact, extensive studies have shown the benefits of smoking cessation on the mortality and morbidity rates in all age groups of smokers [75].

This study had some limitations that should be considered while interpreting the results. First, this study was conducted in an urban area of Iran and the results may not be extrapolated to the whole country and do not necessarily show inequalities in across Iran. Second, this study had a cross-sectional design and therefore the findings do not indicate causality relationships. Moreover, we used a subjective indicator, i.e. SRH, instead of objective indicators like the results of clinical examination or prevalence of chronic conditions. Face-to-face interview for data collection might be sensitive to information bias.

## Conclusion

The results of the present study provide insight to different factors associated with poor SRH, and therefore can be used to develop targeted strategic interventions aiming at QoL improvement. The results showed inequalities in health status between the rich and poor. In other words, people with higher income had a better health status. Therefore, health system planners and policymakers should offer solutions to reduce these inequalities. The main determinants of socioeconomic-related inequalities in health were SES, presence of chronic conditions, physical activity, and BMI. Therefore, designing and implementing proper interventions to improve physical activity in adults as well as prevention and management of chronic conditions can improve the QoL and enhance the health outcomes of adults.

## Data Availability

The data used for the analysis in this study are available from the corresponding author upon reasonable request.
